# Structured Inequality, Uncertain Lifespans: Demographic Perspectives on Predicting Individual-Level Longevity

**DOI:** 10.1111/padr.70065

**Published:** 2026-04-13

**Authors:** CASEY F. BREEN, NATHAN SELTZER

**Affiliations:** Department of Sociology, Population Research Center, and Center for Aging and Population Sciences, University of Texas at Austin, Austin, TX, 78712, USA; Department of Demography, University of California, Berkeley, Berkeley, CA, 94720, USA

## Abstract

There are striking disparities in life expectancy across sociodemographic groups in the United States, shaped by structural forces such as racism, class inequality, and policy environments. To what extent do sociodemographic characteristics structure—or fail to structure—individual lifespans? Using U.S. Census data linked to administrative death records, we assess how well early-adulthood social, economic, and demographic characteristics predict individual lifespan in a cohort of men born in 1910 and observed through their deaths between 1975 and 2005 (N = 121,000). Despite large group-level disparities, we find that sociodemographic characteristics measured in early adulthood explain less than two percent of the overall variation in individual lifespan. These findings reaffirm a central demographic regularity: variance in life expectancy between groups is small compared to variation in lifespan within groups. This highlights the fundamentally nondeterministic nature of how structural inequality shapes individual mortality.

## Introduction

Population researchers have made substantial progress in understanding the contours, disparities, and determinants of mortality in the United States (Elo 2009; Gutin and Hummer 2021; Dowd, Polizzi, and Tilstra 2025). An extensive body of work has applied classic demographic methods to analyze levels, trends, and inequalities in mortality along racial and socioeconomic lines (Wrigley-Field 2025; Schwandt et al. 2021). In parallel, important advances have been made in theorizing the social origins of mortality disparities (Hayward and Gorman 2004; van Raalte 2021; Link and Phelan 1995; Dannefer 2003) and identifying causal determinants of longevity (Fletcher and Noghanibehambari 2024; Cutler, Deaton, and Lleras-Muney 2006; Chetty and Hendren 2018).

Against the backdrop of descriptive, causal, and theoretical work on mortality, the explosion of rich microdata has enabled a new *predictive perspective*. In this study, we apply this perspective to ask: Can observable sociodemographic characteristics—such as education, income, race, and marital status—predict individual-level lifespan? This question tests the limits of social determinism. If lifespan is highly predictable, it would suggest that one’s lifespan is tightly structured by systemic forces (“demography is destiny”). Low predictability would imply that, despite large and well-documented between-group mortality disparities, most lifespan variation remains unexplained by major social or economic factors. This distinction matters for how we think about lifespan inequality: Is mortality governed more by structural inequalities or by stochastic individual variation?

To evaluate this, we analyze linked U.S. Census and Social Security mortality records for over 121,000 men born in 1910, following a single cohort from early adulthood through death to isolate variation in lifespan among individuals observed from a common baseline age. We observe large between-group disparities for this cohort, with gaps in life expectancy at age 65 of nearly three years between those with high and low education. Yet our predictive models only explain 1.3 percent of the variance in lifespan. This highlights a core tension: there are substantial and important between-group disparities, but individual-level variation is great enough that we cannot use these sociodemographic characteristics to accurately predict lifespan for a given person. In other words, predictability is low not because between-group inequality is absent, but because within-group individual variation dominates individual lifespan (Vaupel 1988; Caswell 2023; van Raalte et al. 2012).

Mortality demography, at its core, is concerned with population rates and group-level disparities. What does a predictive perspective add? First, using predictive performance as a diagnostic tool quantifies how much of the variation in lifespan within a cohort is captured by sociodemographic indicators of structural advantage and disadvantage. This approach generalizes traditional decompositions of lifespan variation, which in most empirical applications have examined one categorical covariate at a time, by translating multiple covariates into measures of explanatory power at the individual level. In short, prediction reframes classical decomposition as an inquiry into individual-level explanatory power, leveraging both categorical and continuous characteristics. Second, limited predictability reveals how inequality and uncertainty coexist: stochasticity shapes the overall distribution of lifespans, while structural disparities shift its mean and skewness but remain limited in their ability to determine individual outcomes. Finally, the low predictability of longevity based on sociodemographic characteristics is itself demographically meaningful. It provides empirical insight into a universal form of uncertainty—one that individuals consider when making life decisions (e.g., Will I outlive my retirement savings?). This perspective aligns with the emerging field of uncertainty demography (Trinitapoli 2023), which calls for making uncertainty more central to demographic inquiry and highlights the distinctive capacity of demographic approaches in illuminating the role of uncertainty in social life.^1^

## Background

Social scientists studying mortality have a long-standing interest in describing aggregate disparities in mortality. There are striking class-based (Elo 2009; Montez, Hummer, and Hayward 2012; Chetty et al. 2016), racial (Hummer, Benjamins, and Rogers 2004; Hayward and Heron 1999; Feigenbaum, Muller, and Wrigley-Field 2019; Wrigley-Field 2020), and geographic (Dowd et al. 2024; Montez, Harward, and Wolf 2017) disparities in mortality. While overall longevity has increased over the course of the 20th century (Dowd, Polizzi, and Tilstra 2025), inequality in mortality has also increased over time in the United States along key dimensions (Preston and Elo 1995). Researchers have also documented paradoxical or surprising mortality dynamics, including mortality crossovers (Vaupel, Manton, and Stallard 1979; Wrigley-Field 2020) and the Hispanic mortality paradox (Hummer 2000; Elo et al. 2004; Lariscy, Hummer, and Hayward 2015).

A particularly relevant line of research decomposes variance in longevity into *between-group* and *within-group* contributions (van Raalte et al. 2012; Caswell 2023; Steiner, Tuljapurkar, and Orzack 2010). Between-group variation reflects systematic differences in life expectancy across groups defined by characteristics such as race, class, or geography. This is sometimes referred to as heterogeneity in the mortality literature (Caswell 2023). In contrast, within-group variation captures the differences in lifespan among individuals who share similar risk profiles. This is sometimes referred to as individual stochasticity (Caswell 2009), dynamic heterogeneity (Snyder and Ellner 2018), intragroup heterogeneity (Permanyer, Sasson, and Villavicencio 2023), and luck (Steiner, Tuljapurkar, and Orzack 2010). This is where chance processes, not observed characteristics, drive variation in lifespan among individuals with identical mortality risk profiles.^2^ Both between-group and within-group variation jointly contribute to overall lifespan variation.

This conceptual distinction has motivated recent empirical work quantifying the relative contribution of between-group and within-group variation to overall lifespan variation (Seaman, Riffe, and Caswell 2019; Permanyer et al. 2018). These studies suggest that within-group variation dominates, with characteristics such as income, education, or neighborhood deprivation only explaining a small fraction of overall lifespan variation (Caswell 2023). Most applications of variance decomposition in demography have focused on single categorical factors considered separately; however, recent methodological extensions allow multiple covariates and their interactions to be incorporated within decomposition frameworks (Caswell and Van Daalen 2025). Our approach complements this line of work by shifting from variance partitioning toward individual-level prediction using high-dimensional covariate sets.

### Prior research on mortality prediction

Improvements in data and computation have made individual-level prediction a more feasible research goal (Kashyap 2021), and prediction is increasingly seen as a valuable tool for social science research (Hofman et al. 2021). Several studies have applied a *predictive perspective* to estimate individual mortality (Einav et al. 2018; Rose 2013; Badolato et al. 2026; Savcisens et al. 2024) and to identify the most important predictors of survival (Goldman, Glei, and Weinstein 2016;^2017^; Puterman et al. 2020).^3^ These efforts align with broader methodological shifts in the social sciences and efforts to predict life outcomes (Zheng and Cheng 2025; Salganik et al. 2020).

Studies of individual mortality prediction can be broadly classified as follows: (1) studies that predict all-cause mortality using sociodemographic, behavioral, and health variables from surveys or administrative data; and (2) clinical studies that predict short-term mortality based on diagnoses, symptoms, and other patient-level information.^4^ Here, we focus on studies predicting all-cause mortality (Table 1).

To date, mortality prediction studies have used a period-based design. In this design, researchers pool individuals of different ages and predict their survival over a fixed horizon, addressing questions such as, “Will this individual die in the next five years?” For example, Rose (2013) used data from an aging cohort in Sonoma County to predict five-year mortality from physical activity, smoking, self-rated health, and age, achieving moderate predictive power (*R*^2^ = 0.201). Badolato et al. (2026) used data from the Health and Retirement Study to estimate mortality hazard functions, pooling person-wave observations across survey years. They find overall predictive accuracy to be low, especially for men, non-Hispanic Blacks, and individuals with low education. Across all models, age is by far the strongest predictor.

Even with detailed medical data, mortality prediction efforts report mixed success. Einav et al. (2018) used Medicare claims to predict 12-month mortality for elderly patients and found that even the highest risk individuals had less than a 25 percent chance of dying, suggesting that mortality is difficult to predict even with detailed health information. In a prominent empirical example, Savcisens et al. (2024) used Danish registry data and a large language model to predict premature mortality among adults aged 30–60 over a four-year window. The authors reported mixed success even using age as a covariate, with their best performing model achieving a corrected Matthews correlation coefficient of 0.41, indicating only modest ability to classify deaths.

Period-based approaches are well-suited to many practical forecasting exercises that assess mortality risk across a population heterogeneous in age. Although even the most comprehensive studies have found clear limits to predicting lifespan (Badolato et al. 2026), such predictive mortality models have plausible use cases: for example, identifying individuals at the highest short-term risk of death.^5^ In a period-based design, models generally rely on age as the key predictor. Because mortality risk rises steeply with age, models can achieve moderate predictive performance simply by inferring that older individuals are more likely to die. Even when age is excluded, many covariates (e.g., self-rated health or homeownership status) proxy for age, meaning that much of predictive performance hinges on chronological aging rather than social or structural factors.

In contrast, a cohort perspective offers a cleaner test of how much variation in lifespan measurable characteristics can explain. This perspective asks: “At what age will a given member of a birth cohort die?” By following individuals born in the same year, this design holds age constant and absorbs cohort-level effects, isolating the contribution of sociodemographic factors to within-cohort variation in lifespan. To our knowledge, no prior study has applied a cohort-based prediction framework to quantify lifespan predictability in the United States.^6^ Applying this perspective, we quantify how sociodemographic characteristics structure within-cohort variation in individual longevity.

### Data and study design

In this study, we use the publicly available CenSoc-DMF dataset (Goldstein et al. 2021). This dataset links the full-count U.S. 1940 Census (Ruggles et al. 2020) with mortality records from the Social Security Death Master File (DMF). The DMF captures nearly all deaths in the United States over age 55 between 1975 and 2005 (Alexander 2018; Hill 2001). The 1940 Census provides individual-level sociodemographic characteristics that we use as predictors, including educational attainment, race, income, housing tenure, occupation, and marital status. The CenSoc-DMF file does not include women, as surname changes during marriage preclude reliable record linkage between the 1940 Census and Social Security mortality records. For more technical details on data linkage and methodology, see Breen, Osborne, and Goldstein (2023).

The CenSoc-DMF dataset offers three key advantages for mortality prediction. First, it enables the study of real birth cohorts tracked longitudinally over a 30-year mortality observation window. Second, its large size provides sufficient statistical power to analyze individual birth cohorts. Finally, it includes the core sociodemographic characteristics that are standard indicators of structural inequality.

**FIGURE 1:**
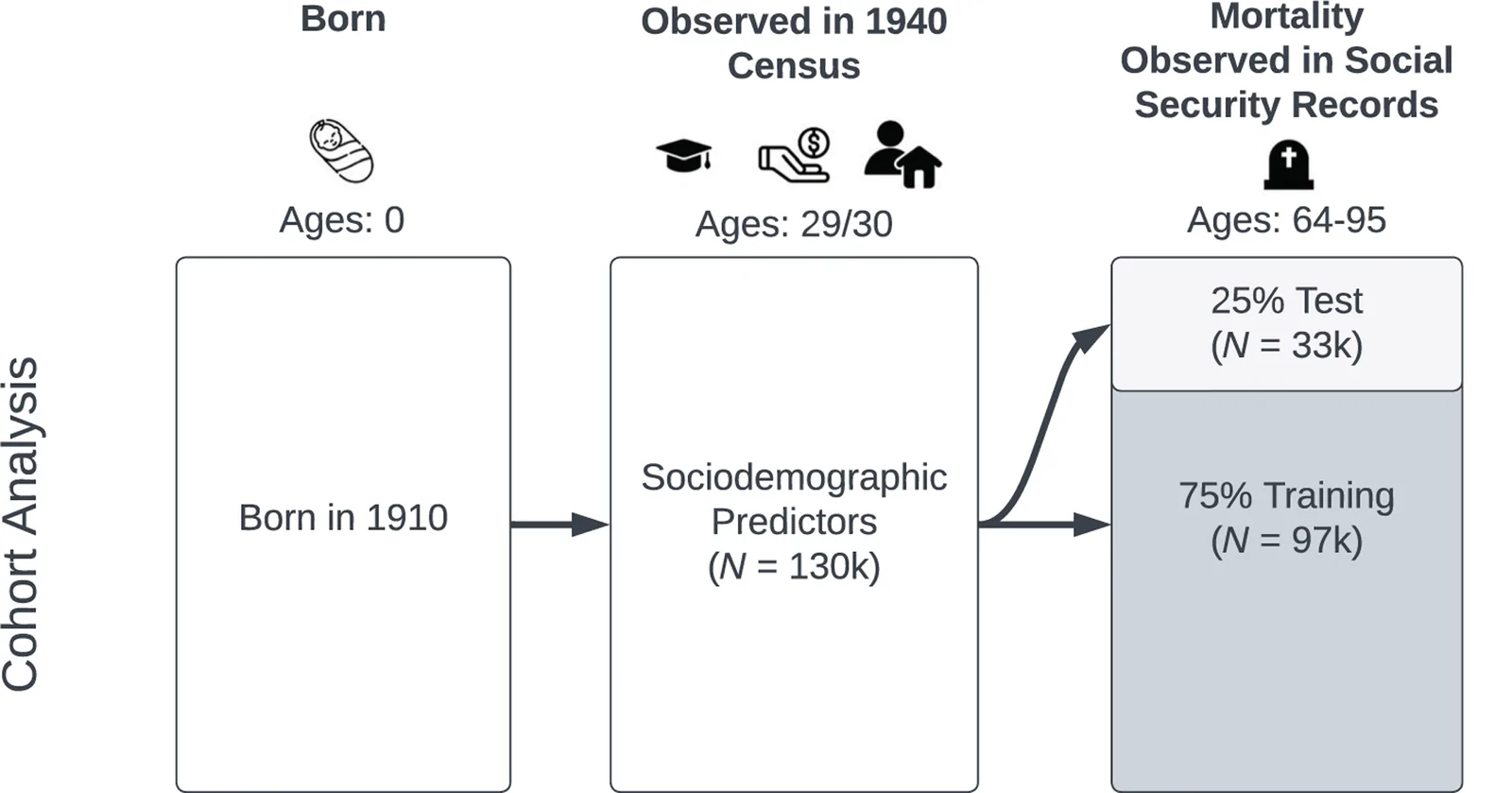
Overview of our analytic sample. We observe their early-adulthood characteristics in the 1940 Census at age 29 or 30, and their mortality between ages 64 and 95

For our analysis, we focus on individuals born in 1910, who were observed in the 1940 Census at age 29 or 30 and died in our mortality observation window between ages 64 and 95. This sample is illustrated in Figure 1. By restricting the analysis to a single birth cohort, we hold age constant and isolate variation in lifespan. We focus on this cohort, who by 1940 had largely finished their education and entered the labor force, offering a stable snapshot of early-adult socioeconomic conditions.

**FIGURE 2:**
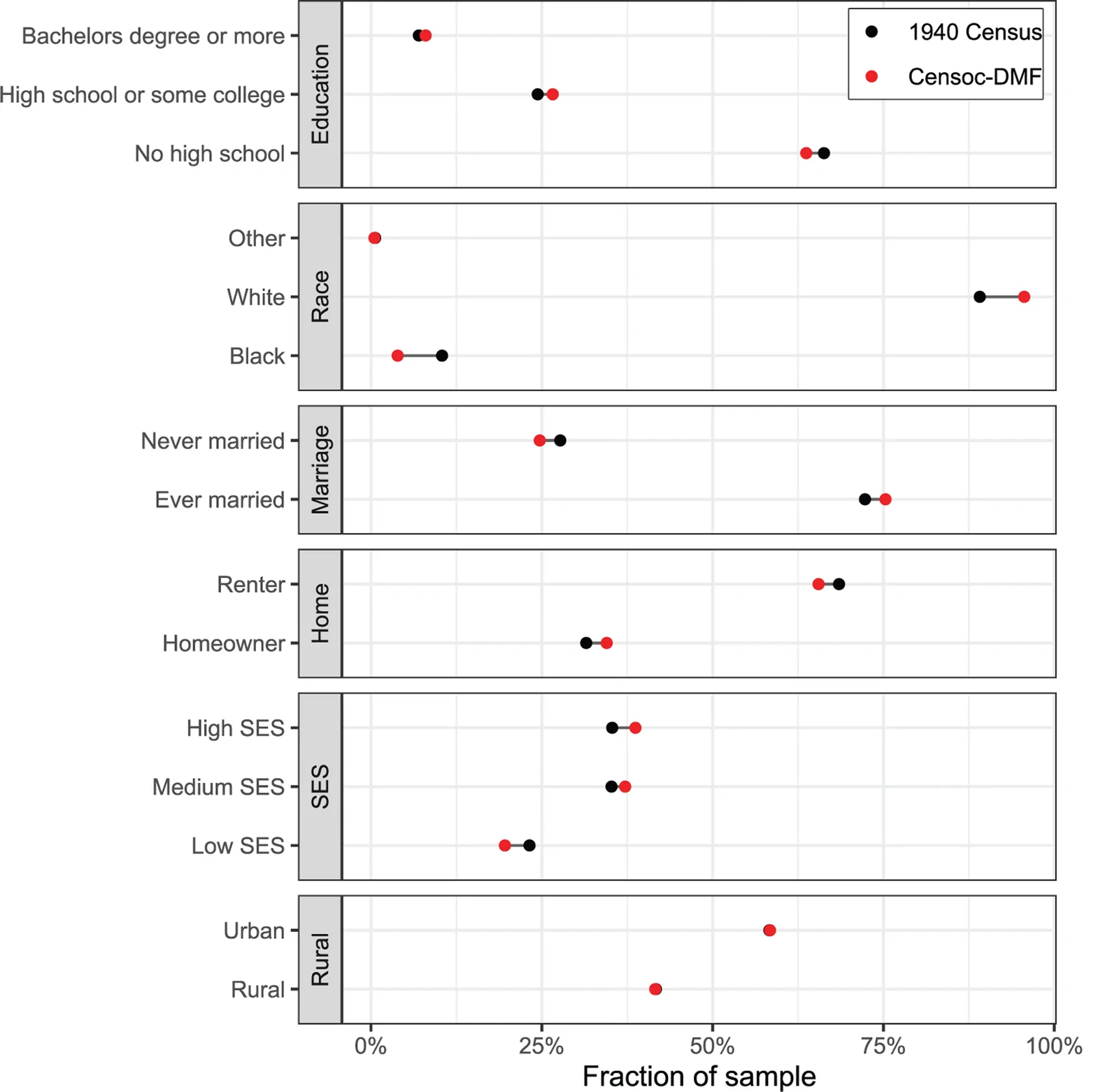
Each facet compares the composition of our analytic sample (red) with that of the 1940 U.S. Census (black) for a given covariate. Overall, the sample composition aligns closely with the population

To assess representativeness, we compare the composition of our linked 1910 birth cohort sample to the composition of all men in the 1940 Census born in 1910. As shown in Figure 2, our linked sample is broadly representative of the corresponding birth cohort observed in the 1940 Census. Like most historical linked samples, it slightly overrepresents individuals of higher socioeconomic status and White individuals. Because our focus is on assessing predictive accuracy, not making population-level inferences, this slight compositional difference is unlikely to affect our results.

All 1940 Census covariates plausibly related to mortality were included as predictors (see Table S1 in the Supporting Information). Categorical covariates (e.g., race, marital status) were dichotomized, and continuous covariates were standardized to a common scale, with a mean of 0 and a standard deviation of 1. The outcome measure is age at death in years, and we exclude cases with missing data.^7^ These predictors are measured cross-sectionally at ages 29 or 30 and do not capture subsequent changes over the life course.

## Methods

We use a machine learning approach to assess how well an individual’s lifespan can be predicted from sociodemographic characteristics. Flexible algorithms are well-suited to this task because they can detect nonlinearities and interactions among correlated predictors (Lundberg, Brand, and Jeon 2022). We implement an ensemble Superlearner (Van der Laan, Polley, and Hubbard 2007), which combines multiple algorithms to improve predictive accuracy and reduce overfitting. Specifically, the Superlearner combines predictions from multiple algorithms, weighting them according to their performance.^8^

We randomly split the sample into a training partition (75 percent) and a holdout partition (25 percent).^9^ The training set includes all predictors along with our outcome of interest, age at death in years. We use this training data to fit the full set of machine learning algorithms. To evaluate predictive performance, we apply the trained algorithms to the holdout set, withholding information on actual ages at death. We then compare the predicted ages at death with the true, withheld outcomes to assess model accuracy. Additional implementation details, including algorithm specifications and validation procedures, are provided in Appendix Section B of the Supporting Information. Our completed REFORM checklist, a reproducibility and transparency framework for machine learning (Kapoor et al. 2024), is included in Appendix Section F of the Supporting Information.

## Results

To contextualize our prediction results, we first examine between-group disparities in longevity for our focal 1910 birth cohort. These descriptive patterns highlight the well-known social gradients in mortality. We then assess how much of the individual-level variation in lifespan can be predicted using machine learning models trained on the same data.

### Between- and within-group differences in longevity.

**FIGURE 3:**
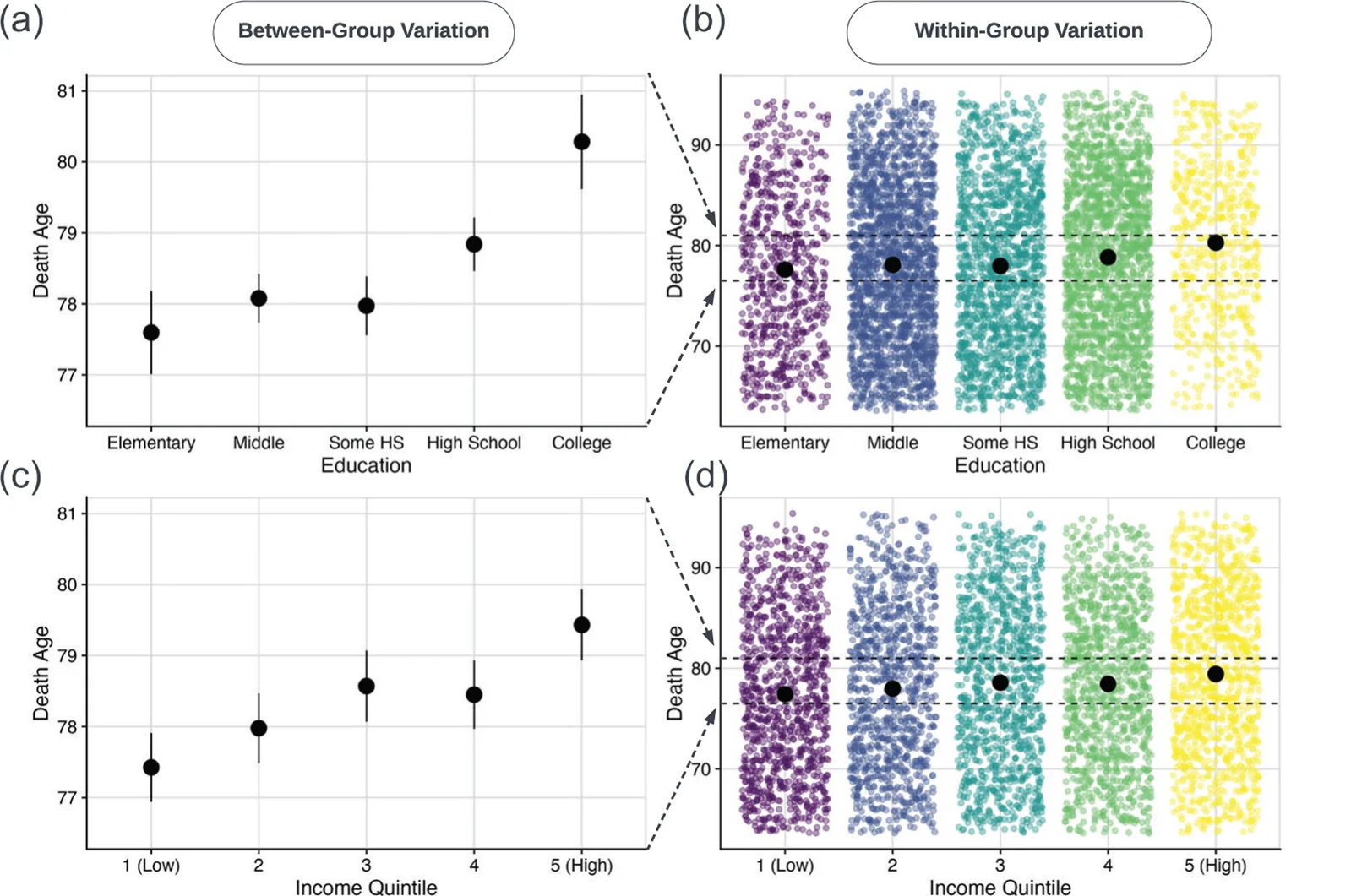
Comparison of within-group and between-group variation for a 5 percent random subsample of the birth cohort of 1910. (a) and (c) Group-level life expectancy across education and income groups. (b) and (d) Group-level life expectancy with individual lifespans overlaid, representing variation in individual outcomes within groups. Individual lifespans are horizontally jittered to improve visibility. Uncertainty bars show 95 percent confidence intervals (not visible in panels c and d because they are smaller than the plotted points)

As shown in Figure 3a, we observe a clear educational gradient in longevity, with higher educational attainment associated with higher life expectancy. We focus on educational attainment as it has a well-established relationship with mortality (Montez and Bisesti 2024) and is a common proxy for social class in the United States (Muller and Roehrkasse 2022; Pettit and Western 2004). A similar pattern is observed for income (Figure 3c), with higher wage and salary income associated with longer life expectancy. These between-group differences in life expectancy align in direction and approximate magnitude with other empirical studies (Halpern-Manners et al. 2020; Lleras-Muney, Price, and Yue 2020). These group-level differences reflect the between-group component of lifespan variation.

In Figures 3b and 3d, we present the same group-level estimates overlaid with individual-level death ages (represented by dots), which illustrate the within-group component of lifespan variation. The between-group differences in life expectancy are substantial: those with college degrees lived nearly three years longer, on average, than those with only elementary education. However, these differences are small relative to the individual-level variation within groups. Many college graduates die before age 70, while many with only an elementary education live past age 90.

### Individual-level predictions.

**FIGURE 4:**
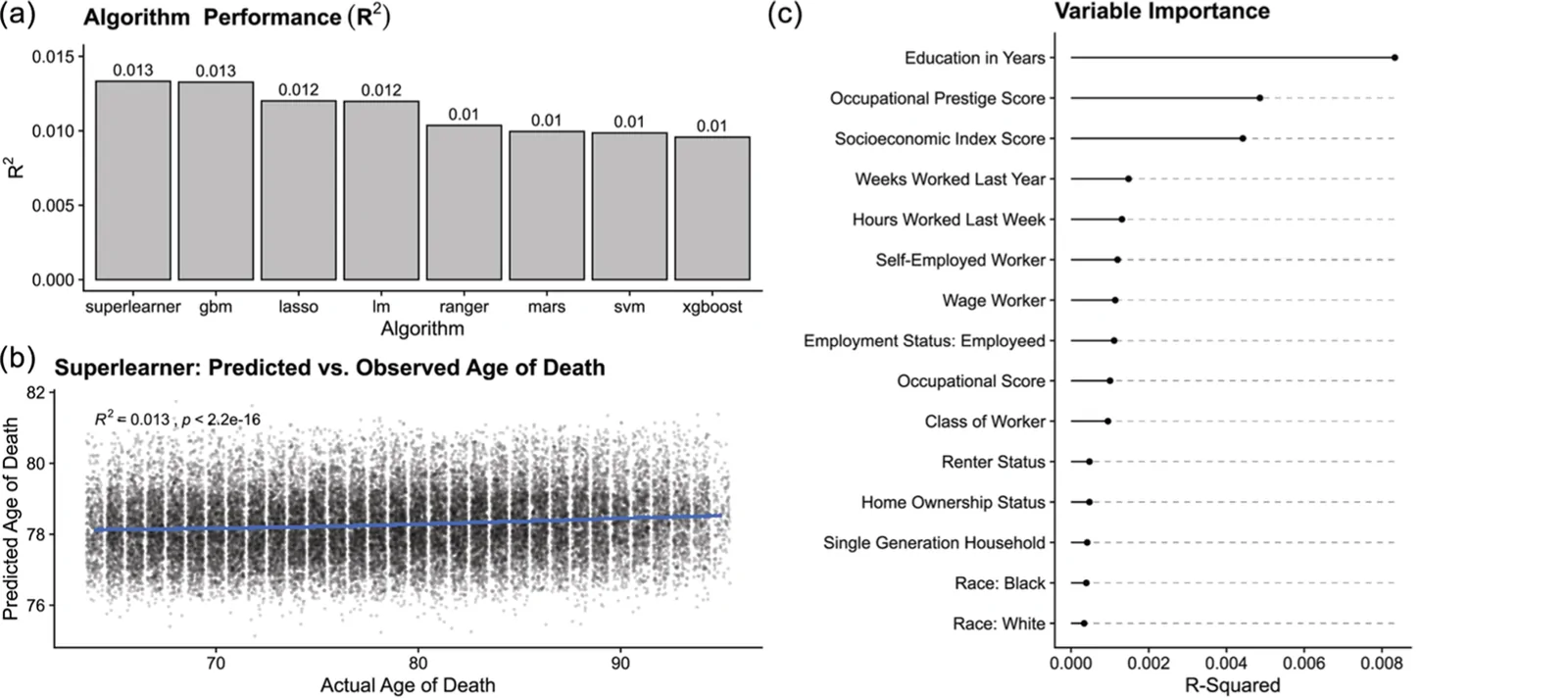
(a) *R*^2^ value for each machine learning algorithm. (b) Predicted versus observed values from the Superlearner algorithm. (c) Relative importance of the top 15 predictors.

We next assess how well our machine learning models predict individual lifespan. As shown in Figure 4a, none of the algorithms are able to accurately predict lifespan. The Superlearner algorithm—a weighted ensemble of the other algorithms—had the best out-of-sample performance in our holdout set (*R*^2^ = 0.013). This low *R*^2^ indicates that our best performing algorithm offers little improvement over simply predicting the sample mean age at death.

Figure 4b plots the correlation between the predicted age of death and the true, withheld age of death for the holdout sample. There is a very weak correlation between our predicted and observed age of death (*R* = 0.114). These results suggest that, even with core sociodemographic predictors and flexible algorithms, most of the variation in individual lifespan remains unexplained. Further, most of the predictions are narrowly concentrated between the ages of 76–82, indicating that the models largely fail to distinguish between individuals who die relatively early and those who live to older ages.

We next examine which predictors contribute most to the limited variation the model captures. Figure 4c plots variable importance for the top-performing Superlearner algorithm. Variable importance can be defined in many ways, each providing different insights into how an algorithm relies on a given variable for its predictions. We calculate variable importance as the *R*^2^ of each predictor from a univariate regression (for an alternative permutation-based variable importance, see Figure S5 in the Supporting Information). Education in years and occupational prestige score^10^ were the two most important predictors, aligning with theoretical expectations (Galea et al. 2011; Montez and Bisesti 2024; Elo 2009).

We conducted several supplementary analyses. First, we examined predictability in a dataset that includes women, albeit with a shorter mortality observation window of 1988–2005. We found similarly low predictive accuracy for this sample (*R*^2^ = 0.012). Although the shorter window limits comparability with the main results, this finding suggests that lifespan is likewise difficult to predict among women (Appendix Figure S1 of the Supporting Information). Second, we assessed predictive accuracy separately for each birth cohort from 1900 to 1920 (Appendix Figure S6 of the Supporting Information), finding that both earlier and later cohorts exhibited similarly low predictive accuracy. Finally, we evaluated predictive accuracy by race and socioeconomic status, finding that our models explained a smaller share of lifespan variance among lower education and lower income groups, as well as among Black Americans (Appendix Figure S7 of the Supporting Information). Given the large sample sizes, these likely reflect true greater underlying uncertainty in lifespan for these subgroups.

## Discussion

The key methodological innovation of our study is that we make predictions within a cohort framework, focusing on a single birth cohort, rather than pooling individuals across ages. This design allows us to isolate the predictive power of sociodemographic characteristics net of age. We find that, despite large between-group differences in longevity, these sociodemographic characteristics explain less than 2 percent of the overall variation in lifespan. These results offer a new empirical lens into a well-established demographic fact: variation in mortality risk across groups is much smaller than the variation in lifespan within those groups (Vaupel 1988; Caswell 2009;^2023^). Theoretically, our findings center uncertainty as a fundamental feature of the human lifespan, highlighting that individual lifespan is only weakly determined by observable sociodemographic characteristics and shaped in large part by stochastic processes operating within structured social contexts.

These findings also echo classical results from the frailty modeling tradition. Vaupel (1988) empirically documented how the lifespans of parents and children are only weakly correlated, despite the intergenerational transmission of both genetic and environmental factors. Using simulation, Vaupel (1988) shows that even if frailty is directly inherited from parents, it still explains only 2–5 percent of the total variance in lifespan.^11^ The limited explanatory power of socioeconomic factors, therefore, aligns with the broader demographic insight that individual longevity is shaped only marginally by systematic determinants and largely by stochasticity.

Our results also add to a growing literature on the limits of prediction in other social domains (Salganik et al. 2020; Arpino, Le Moglie, and Mencarini 2022; Dressel and Farid 2018; Zheng and Cheng 2025). Comparisons across prediction exercises are inevitably limited by differences in data, features, and modeling approaches. Nonetheless, we situate our findings against those from other studies as illustrative points of reference. Rather than offering strict performance benchmarks, these results provide context for the scale of predictability we observed. For instance, Zheng and Cheng (2025) predict midlife socioeconomic status using 4,000 covariates from the stratification literature, finding a relatively high *R*^2^ of 0.5. Compared with the life outcome prediction results in Salganik et al. (2020), our reported *R*^2^ is lower than material hardship at age 15 (*R*^2^ = 0.23), while primary caregiver layoff (*R*^2^ = 0.03) represents a similarly low level of predictability. These comparisons reinforce that lifespan is a unique biosocial outcome shaped by both social and biological processes, with substantial stochasticity that limits predictive accuracy.

This low predictability can also be interpreted through the framework introduced by Lundberg et al. (2024), which decomposes prediction error for life outcomes into two components: learning error and irreducible error. Learning error reflects limitations in model fitting and sample size, while irreducible error captures within-group variance. In our setting, learning error is relatively small due to our large sample, while irreducible error is substantial: even within sociodemographic groups, variation in age of death remains high. This decomposition highlights that most variation in individual longevity remains unexplained by observed sociodemographic factors, reflecting an irreducible component of uncertainty.

On the one hand, the predictive power of observed covariates for longevity is limited; on the other hand, between-group inequalities in mortality remain substantial. Large differences in life expectancy across sociodemographic groups—by race, education, income, or place—can coexist with considerable within-group variation. Such disparities reflect structural inequality, policy-relevant gradients, and historically rooted disadvantage that remain central to mortality demography. Low predictive power should therefore not be equated with low structural inequality; rather, it reinforces mortality demography’s focus on group-level outcomes and highlights the value of frameworks that integrate within- and between-group components to better understand lifespan inequality (Permanyer, Sasson, and Villavicencio 2023; Shi 2022).

Limited predictability clarifies how sociodemographic factors operate at the individual level. Sociodemographic characteristics may explain only a small share of individual-level lifespan variance while still generating meaningful differences in mortality risk and enabling discrimination between individuals’ relative survival prospects, especially when age is considered (Badolato et al. 2026). From this perspective, predictive analyses complement rather than replace traditional approaches by highlighting how structural gradients and individual-level uncertainty coexist. Prediction provides a quantitative lens for assessing the scale at which social determinants determine lifespan without implying deterministic life-course trajectories. Simply put, individual-level lifespan predictability is low because the between-group inequality is dominated by within-group variation (van Raalte et al. 2012; Vaupel 1988; Caswell 2023). This does not diminish the crucial importance of studying between-group disparities, which remain central and policy-relevant.

These findings can also be situated within the emerging field of uncertainty demography, which treats uncertainty as an inherent component of population processes deserving of study in its own right (Trinitapoli 2023). Our analysis extends this perspective to mortality by interpreting the predictability of individual lifespans as a measurable form of population-level uncertainty. Low predictability reflects the stochasticity inherent in mortality (uncertainty as an outcome), while that stochasticity is shaped by macrolevel forces such as economic volatility, environmental shocks, epidemics, and what might be called life luck—forces that generate uncertainty as a cause of mortality variation. In this sense, uncertainty is not merely noise to be explained away by better covariates and models, but a defining demographic feature that structures both individual and collective experiences of life and death. More broadly, this stochasticity is a key feature not only of lifespan variation but also outcomes such as morbidity and lifetime reproductive output, where variance itself is a substantive object of analysis (Caswell 2023; van Daalen et al. 2022).

Building on this uncertainty demography perspective, our findings highlight how limited predictive performance can coexist with persistent structural inequalities. Rather than viewing limited predictability as a limitation, we interpret it as empirical evidence for a nature–nurture–chance framework, which calls for greater attention to the role of chance in shaping individual lifespans (Sasson 2025). This is operationalized here through high predictive uncertainty in cohort lifespan outcomes: prediction offers a complementary lens for assessing the balance between systematic social determinants and residual uncertainty. This aligns with evidence from studies of genetically identical twins, which find that substantial variability in longevity persists even when genetic and environmental factors are closely aligned (Finch and Kirkwood 2000).

This lifespan uncertainty has meaningful implications for lived experiences. Expectations about survival influence human capital investments (Sasson 2016), investment strategies (Abel 1985; Barro and Friedman 1977), and health and fertility decisions (Picone, Sloan, and Taylor 2004; Nettle 2010). Although our study does not directly examine behavioral responses to uncertainty or perception thereof, the stratified patterns of predictability we document suggest that lifetime uncertainty is unevenly distributed across populations. Further, lifespan uncertainty may be especially pronounced and salient in violent or high-risk contexts, where mortality risks both shorten lives and increase unpredictability in the timing of death, complicating long-term planning and decision-making (Aburto et al. 2023).

Several limitations and opportunities for future research warrant discussion. Our main analysis is restricted to men born in 1910, observed in the 1940 Census, and dying between ages 65 and 95. While this design enables a cohort-based approach to lifespan prediction, it excludes early deaths before age 65; nevertheless, approximately 65 percent of this cohort died within the observed window (Breen and Osborne 2022). Our predictors are measured cross-sectionally in early adulthood at age 29 or 30, and certain predictors, such as income, occupation, or marital status, may change over the life course. As a result, we cannot assess how life course trajectories or covariates measured at older ages might alter predictability, even though such measures could plausibly carry stronger mortality signals (e.g., marital dissolution in later life). Longitudinal life-course data may improve predictive performance relative to our baseline; however, even substantial improvement—for example, a doubling or tripling of explanatory power—would still imply relatively low overall predictability. Moreover, several key predictors, including educational attainment, are largely fixed by early adulthood for this cohort.

By design, we focused on sociodemographic variables that capture systemic inequality. Biological and behavioral predictors are not the primary focus of this study, which centers on the social structuring of mortality. Such predictors do, however, overlap with the sociodemographic characteristics considered here, and future work could explicitly consider these predictors.

Our primary analysis focuses on the cohort of 1910 and may not generalize to other more recent birth cohorts. Replication efforts with cohorts born between 1900 and 1920 revealed similarly low predictability (*R*^2^ values ranging from 0.5 percent to 1.4 percent). Our main analysis is also focused on men due to data constraints: surname changes for women at marriage precluded their linkage between the 1940 Census and mortality records. However, in Appendix Section C of the Supporting Information, we replicate our analysis on a similar linked file that includes a shorter mortality observation window but includes both genders. We find similarly low predictability in this sample with both genders (*R*^2^ = 0.012).

Taken together, our findings highlight the limits of sociodemographic characteristics in structuring one of life’s most consequential outcomes: how long one will live. Structural inequality generates large and meaningful differences in life expectancy across groups, yet these same characteristics explain little of the variation in lifespan among individuals. This underscores a central tension: while between-group disparities are substantial and socially significant, the within-group variation dominates individual lifespans. This demonstrates the fundamentally nondeterministic nature of how structural inequality shapes individual lifespan.

## Supplementary Material

Supplementary Material

## Figures and Tables

**TABLE 1:** Summary of past studies on individual-level prediction of all-cause mortality

Study	Data source	Covariates	Sample size

Puterman et al. (2020)	Health and Retirement Study	Sociodemographic, behavioral, and health	39,248
Badolato et al. (2026)	Health and Retirement Study	Sociodemographic, behavioral, and health	39,248
Savcisens et al. (2024)	Danish Registry Data	Sociodemographic, behavioral, and health	100,000
Rose (2013)	Study of Physical Performance and Age-Related Changes in Sonoma	Sociodemographic, behavioral, and health	2,092
Einav et al. (2018)	Medicare enrollees	Sociodemographic, behavioral, and electronic medical records	5,631,168
This study	CenSoc (linked census and Social Security Mortality records)	Sociodemographic	121,000

## Footnotes

1 Trinitapoli (2023) frame uncertainty demography as treating uncertainty not as the amount of error in an estimate but as a constitutive feature of social and demographic life. It examines how populations experience, reproduce, and navigate uncertainty as a social fact.

2 This stochastic variation may reflect truly random processes or latent individual-level determinants. What appears as “luck” in lifespan may instead reflect fine-grained variation in exposures, behaviors, genetics, or life events that are unmeasured. This differs from variance calculated from the rates estimated for groups using life-table or Markov formulations, which assume homogeneity within groups by construction (Caswell 2023).

3 A strength of the predictive approach is its ability to identify the strongest individual-level predictors of mortality. Goldman, Glei, and Weinstein (2016);^2017^) found that self-rated health, mobility limitations, and difficulties with instrumental activities of daily living consistently outperformed clinical measures like obesity or diabetes in predicting survival. Puterman et al. (2020) found the most important predictors of longevity included smoking behavior, alcohol abuse, and history of divorce.

4 For example, Ottenhoff et al. (2021) predicted 21-day mortality for hospitalized COVID-19 patients using sociodemographic and clinical data. While these models perform reasonably well for short time horizons, they rely on detailed clinical data not typically available in population datasets.

5 We cannot rule out the possibility that model performance will improve in the future. As Yan and Rahal (2025) notes, the predictability of social systems remains uncertain: data and algorithms continue to improve, and claims of “unpredictability” are context dependent.

6 Prior work has examined cohort patterns in lifespan variance and frailty in several European countries for historical cohorts, finding that only a small amount of lifespan variance is due to frailty (Hartemink, Missov, and Caswell 2017).

7 Approximately 9 percent of records had at least one missing value. Imputing these missing values using multiple imputation, rather than dropping cases with missing values, produced comparable but slightly lower predictive performance.

8 The motivation behind the ensemble Superlearner approach is that a weighted combination of different algorithms generally outperforms any single algorithm. The ensemble Superlearner algorithm selects the optimal weighted combination using a cross-validation procedure within the training set to minimize overfitting risk (Phillips et al. 2023). For our ensemble Superlearner, we include a set of widely used machine learning algorithms: random forests, linear regression, gradient boosting machines, lasso regression, extreme gradient boosting machines, and support vector machines.

9 We chose a classic train/test split over *k*-fold cross-validation due to computational limitations of our large sample. Given the large sample size, a single holdout set is sufficient for reliable out-of-sample evaluation while avoiding the prohibitive computational cost of repeatedly reestimating ensemble models required by *k*-fold cross-validation.

10 We use the Siegel occupational prestige score, which calculates an occupational prestige score for each occupation based on perceived status from a survey of the general population.

11 In this exercise by Vaupel (1988), frailty is represented by a single parameter that aggregates all mortality risk. In the simulations, each individual receives a parameter value, which functions as a stand-in for their relative risk in a proportional hazards model.
