# Supplementary material for: Structured Inequality, Uncertain Lifespans: Demographic Perspectives on Predicting Individual-Level Longevity
Source: Popul Dev Rev. Author manuscript; Available in PMC 2026 Aug 29. (PMC13524427; doi:10.1111/padr.70065)
Supplement: Supplementary file 1 [file NIHMS2183517-supplement-Supplementary_Material.pdf]

Supplemental Materials

Structured Inequality, Uncertain Lifespans: Demographic Perspectives on Predicting Individual-level Longevity

Casey F. Breen and Nathan Seltzer

A List of predictors

All predictors for our analyses come from the complete-count 1940 Census. The full set of predictors is shown below in Table A1. We recoded categorical variables into binary (0/1) indicators and standardized continuous predictors to a common scale, with a mean of 0 and a standard deviation of 1.

Variable	Description
Education (years)	Educational attainment in years.
Wage and salary income	Wage and salary income for employees, topcoded at $5,001
Socioeconomic Index Score	Duncan Socioeconomic Index, a composite score reflecting occupational status and earnings
Occupation Income Score	Occupational income score based on median income of all people with a given occupation in 1950.
Household size	Number of persons living in a household, topcoded at 20.
Occupation Prestige Score	Occupational prestige score, based on Siegel's method.
Race: White	Binary variable for whether an individual was classified as White.
Race: Black	Binary variable for whether an individual was classified as Black.
Race: Other	Binary variable for whether an individual was not classified as Black or White.
Metro	Binary variable indicating lives in a metro area.
City	Binary variable indicating whether an individual lived in a city.

Suburb	Binary variable indicating whether an individual lived in a suburb.
Marital Status: Married	Binary variable indicating whether an individual is married.
Marital Status: Divorced	Binary variable indicating whether an individual is divorced.
Marital Status: Widowed	Binary variable indicating whether an individual is widowed.
Marital Status: Single	Binary variable indicating whether an individual is single.
Self-Employed	Binary indicator on whether an individual is self-employed.
Wage Worker	Binary indicator on whether an individual is a wage and salary workers.
Lives with Mother	Binary indicator on whether someone lives in the same household as their mother
Migration: No movement	Binary variable indicating no movement from house in the last 5 years.
Migration: Internal	Binary variable for individuals who moved states in the last 5 years.
Migration International: International	Binary variable for individuals who moved countries in the last 5 years.
Urban	Binary variable distinguishing urban versus rural place of residence.
Labor force Participation	Binary variable indicating labor force participation status.
Farm	Binary variable indicating if the residence is on a farm.
Homeowner	Binary variable indicating homeownership vs. renter status.
Household: Single Generation	Binary variable indicating a single generation household.
Household: Second Generation	Binary variable indicating a two-generation household.
Household: Third Generation	Binary variable indicating a three-generation household.
Employment Status	Binary employment status variable for employed individuals.
State	State of residence in 1940.

Table A1: Variables used for prediction

B Machine Learning Approach

For most prediction tasks, it is impossible to know in advance which predictive algorithm will have the best performance. To overcome this, we use Superlearning. Superlearning—also known as weighted ensembling or model stacking—is a statistical technique for prediction that combines many machine learning algorithms into a single algorithm (Van der Laan, Polley and Hubbard, 2007). The ensemble Superlearner algorithm finds the best weighted combination of algorithms using a k-fold cross-validation procedure to minimize cross-validated risk (Van der Laan, Polley and Hubbard, 2007). In the Superlearning framework, a metalearner is used to combine the predictions of the discrete algorithms.[13] This metalearner is trained exclusively on the training data, using the outputs of the base models as input features.

The motivation behind Superlearning is that a weighted combination of many different algorithms may outperform any single algorithm by smoothing out the limitations of any specific model (Phillips et al., 2023). This avoids the issue of picking an algorithm a priori by selecting the best weighted combination of algorithms using a pre-defined set of decision rules that minimize cross-validated mean squared error.

We fit the Superlearner algorithm on our training data using the following implementation:

1. Split the data into $k = 10$ different folds (partitions) for k-fold cross-validation.

2. Using the first fold as the holdout data, fit each discrete algorithm on the 9 other folds. Make predictions on the holdout fold using each algorithm. Repeat this process for

[13] Here, we use a non-negative least squares regression model as our metalearner.

each fold, using a different fold as the holdout data.

3. Choose a loss function (e.g., mean squared error) and compute a metalearner. The metalearner is a principled approach to combining multiple machine learning algorithms by fitting a regression of outcome variables on the predicted variables to minimize the cross-validated risk of the set of potential weighted combinations.

4. Fit each of the algorithms on the full data.

In addition to asymptotically producing a weighted algorithm that theoretically performs as well or better than the top algorithm, the Superlearner has the added advantage of training many machine learning algorithms at the same time. This allows us to assess the performance of many different machine learning algorithms with an a priori specified evaluation criterion: minimum cross-validated mean squared error. This is a conservative criterion that prioritizes minimizing large predictive errors.

Algorithm	Description	Cross-validated Risk	Superlearner Coefficient
gbm	Gradient boosting machines	63.61	0.47
lm	Linear model	63.63	0.09
xgboost	Extreme gradient boosting	63.89	0.07
ranger	Random forest regression	63.87	0.14
svm	Support vector machine	63.76	0.23
lasso	Lasso regression	63.88	0.00
mars	Multivariate adaptive regression splines	63.63	0.00
mean	Arithmetic mean	64.43	0.00
Superlearner	Ensemble Superlearner	63.59	—

Table A2: Full set of algorithms included in the Superlearner ensemble model for cohort analysis. The cross-validated risk refers to the risk calculated by the Superlearner, the cross-validated mean squared error. The coefficient gives the total weight (contribution) of each algorithm toward the full Superlearner ensemble model.

Table A2 shows the full set of machine learning algorithms used in our Superlearner.

The cross-validated risk is lowest for the gradient boosting machines and highest for the lasso regression and extreme gradient boosting. The Superlearner algorithm most heavily weighted the gradient boosting machines algorithm and the support vector machine algorithm.

B.1 Assessing model performance

Our primary metric for assessing predictability is $R^2_{holdout}$, defined as:

$$R^2_{holdout} = 1 - \frac{\sum_{i=1}^{n}(y_i - \hat{y}_i)^2}{\sum_{i=1}^{n}(y_i - \bar{y})^2} \tag{1}$$

where y_i is the observed age of death in the holdout dataset, $\hat{y}_i$ is the predicted age of death in the holdout dataset, $\bar{y}$ is the mean observed age of death in the holdout dataset, and n is the number of observations. This is a continuous metric that ranges from 0, which represents predictions no better than the mean of the holdout dataset, to 1, indicating perfect predictions. We use this metric to facilitate comparisons with other studies (Salganik et al., 2020; Puterman et al., 2020; Zheng and Cheng, 2025).

As a second measure of predictive performance, we calculate the mean absolute error, the average of the absolute differences between the predicted and true lifespan in our holdout sample. The mean absolute error is defined as:

$$\text{MAE} = \frac{1}{n}\sum_{i=1}^{n}|y_i - \hat{y}_i| \tag{2}$$

where y_i is the observed age of death for individual i in the holdout dataset, $\hat{y}_i$ is the predicted age of death for individual i in the holdout dataset, and n is the total number of observations.

This intuitive metric provides insight into how close, on average, our predicted lifespans are to the true lifespans.

C Prediction for women

For our main analysis, we use the CenSoc-DMF, which includes deaths for the window of 1975–2005 but is restricted to men. In this supplementary analysis, we replicate our main prediction exercise using data from the CenSoc-Numident, which links the 1940 Census with mortality records from the Social Security Numident (Goldstein et al., 2021). In contrast with the CenSoc-DMF, the CenSoc-Numident only includes deaths for a shorter window, between 1988–2005. However, the CenSoc-Numident file includes women, allowing us to replicate our analysis on a dataset that includes women.

Results from this replication are shown in Figure A1. The findings are comparable to our CenSoc-DMF results: all models have very low predictive accuracy. The top-performing Superlearner model explains only 1.2% of the variation in age at death.

D Period perspective

While our main analysis predicts age at death from a cohort perspective, we also conduct a period-based analysis to test the predictability of shorter-term mortality risk of individuals of different ages. Specifically, we predict 5-year mortality using a random sample of 100,000 men aged 54–95 in 1975. This design resembles a cross-sectional survey with follow-up, allowing us to include age as a predictor.

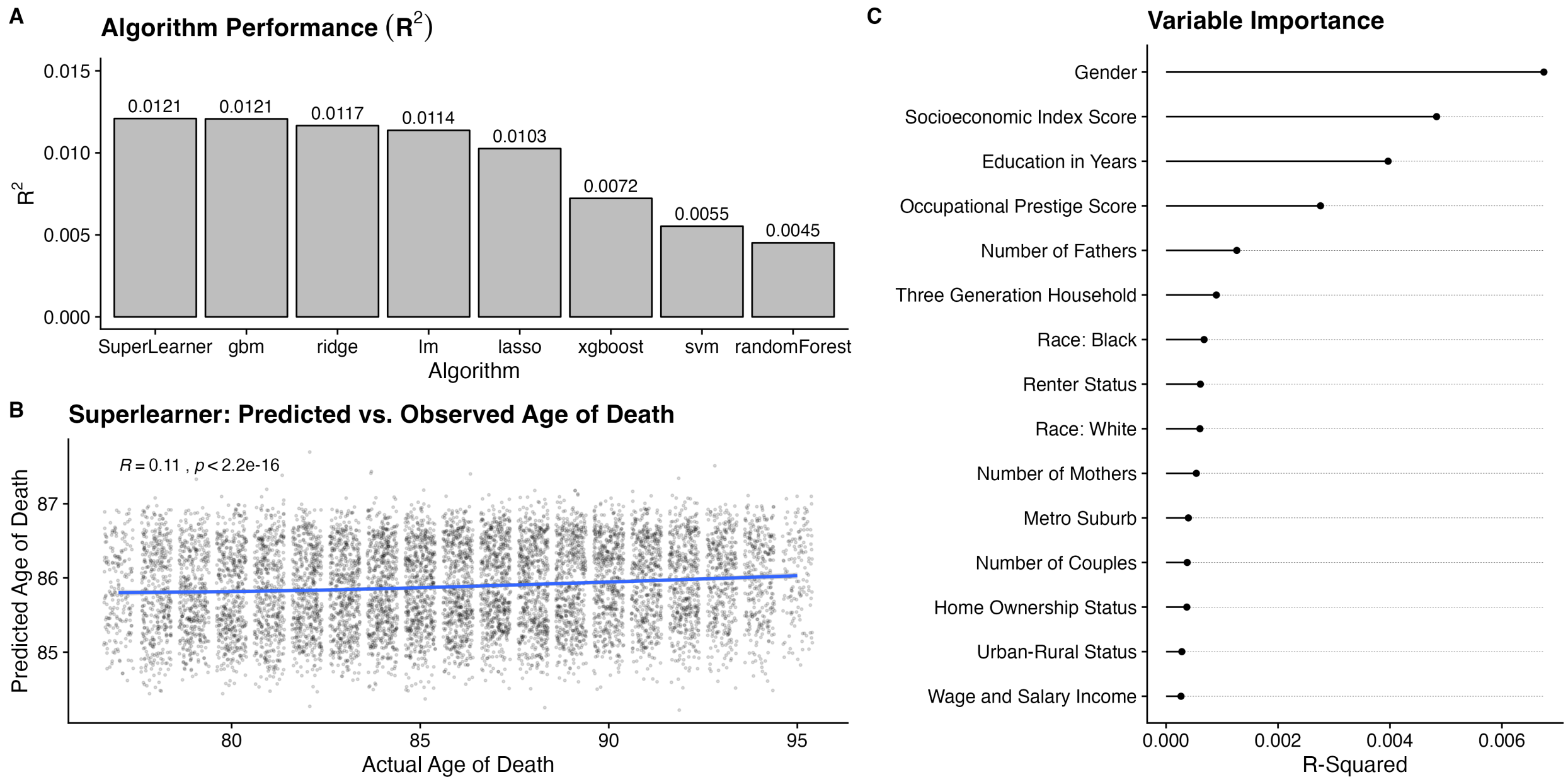

Figure A1: *(A)* R^2 value for each machine learning algorithm. *(B)* Predicted vs. observed values from Superlearner algorithm. *(C)* Relative importance of the top 15 predictors. The CenSoc-Numident cohort of 1910 includes 75,238 individuals (20,167 women and 55,071 men).

We use the same set of sociodemographic predictors as in the cohort analysis with the addition of age. Instead of predicting lifespan, we model a binary outcome: whether an individual dies in a 5-year window between 1975 and 1979. We fit a Superlearner ensemble using algorithms appropriate for classification tasks: logistic regression, elastic net, ridge regression, lasso, random forest, and extreme gradient boosting.

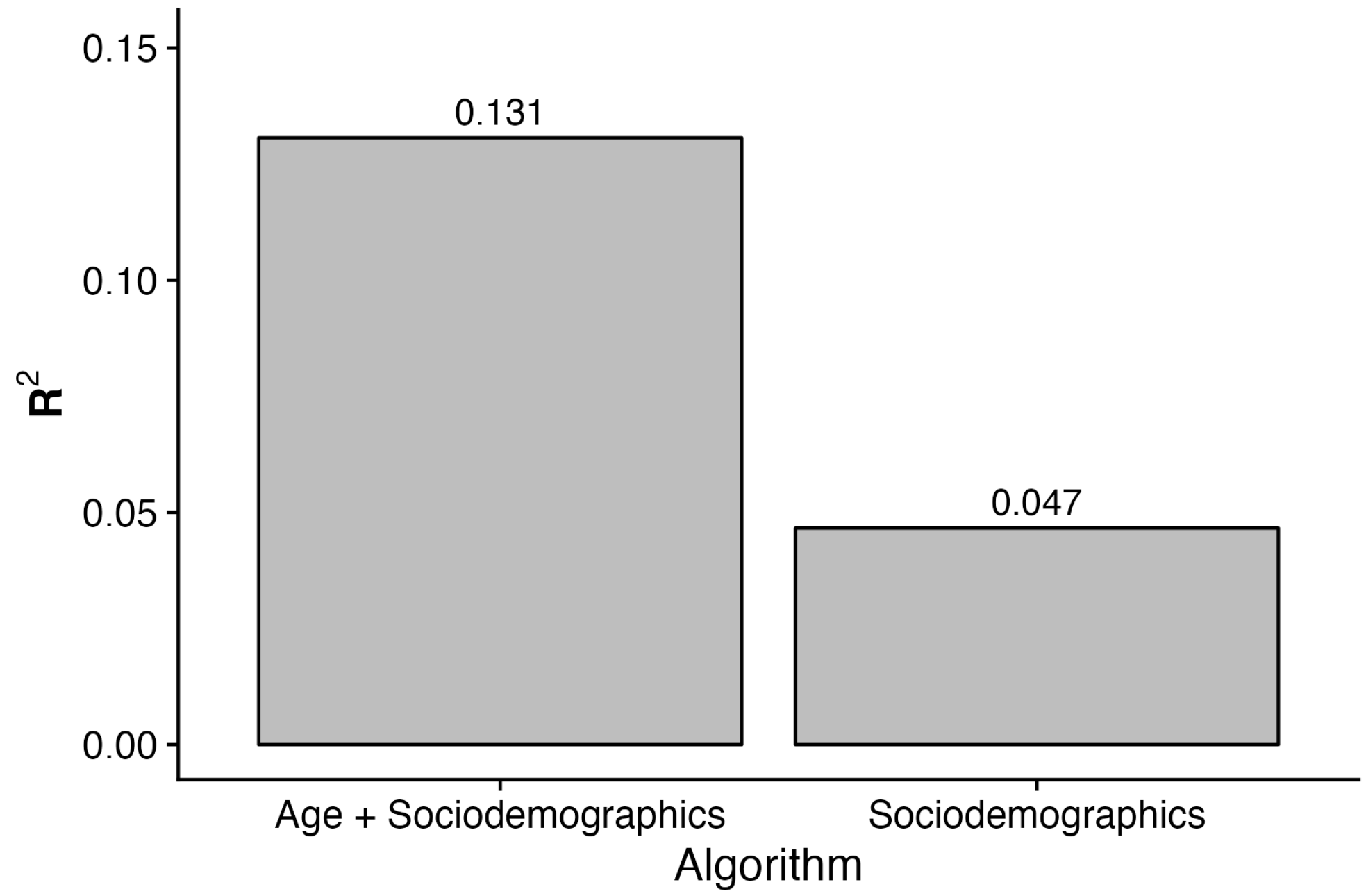

Figure A2: Period analysis results. R^2 for model using age and sociodemographic predictors and models using sociodemographic predictors alone.

Figure A2 shows that the model using both age and sociodemographic predictors achieves modest accuracy ($R^2 = 0.131$). A model trained exclusively on sociodemographics without age performs substantially worse ($R^2 = 0.047$). This suggests limited incremental predictive value from early-life sociodemographic factors. This analysis also reinforces that predicting mortality in the next 5 years results in higher predictive accuracy than using the same characteristics to predict lifespan in a cohort framework.

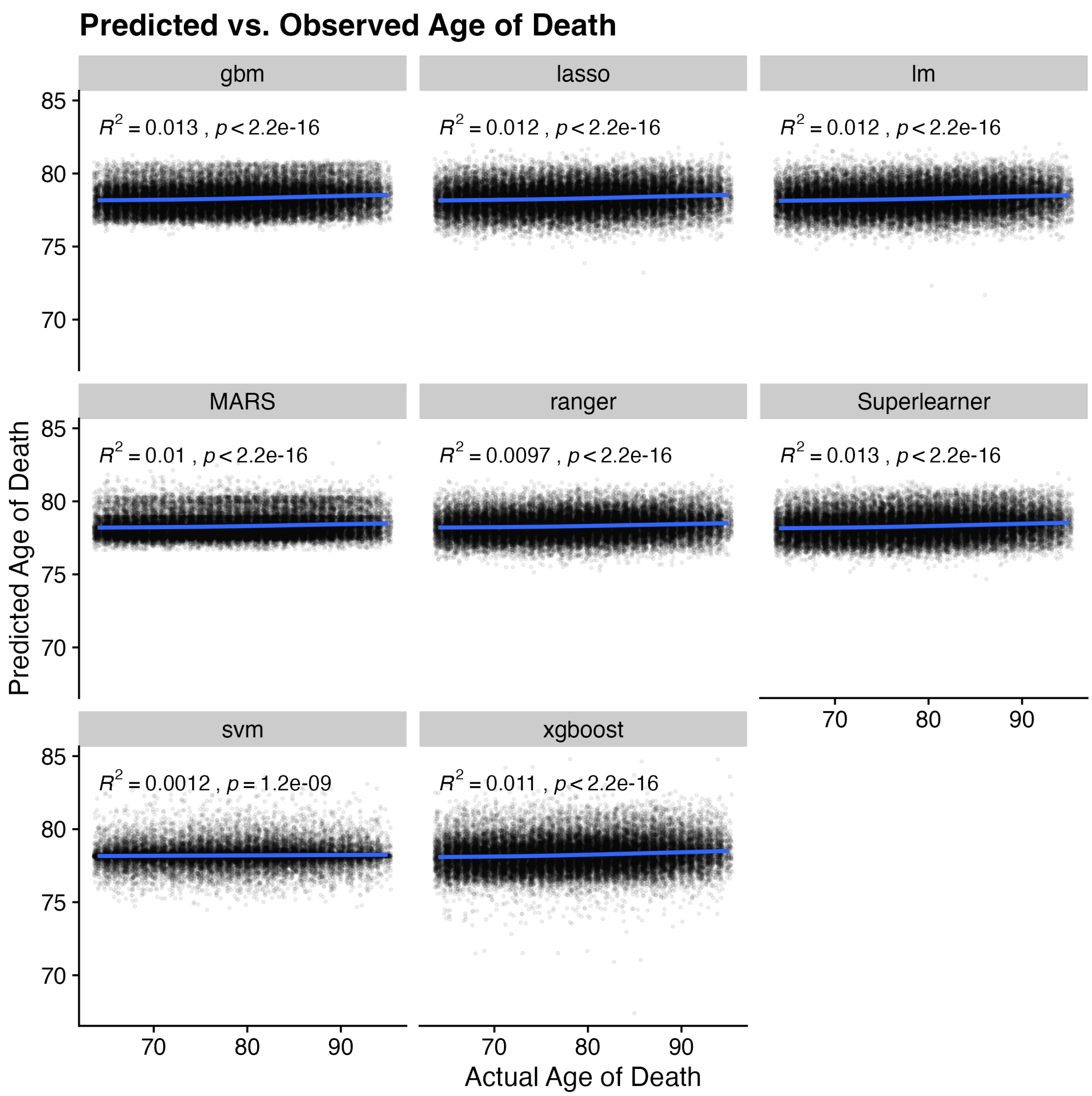

Figure A3: Scatterplots showing predicted vs. observed age of death for eight separate machine learning algorithms used in our main analysis of the cohort of 1910.

E Additional Results

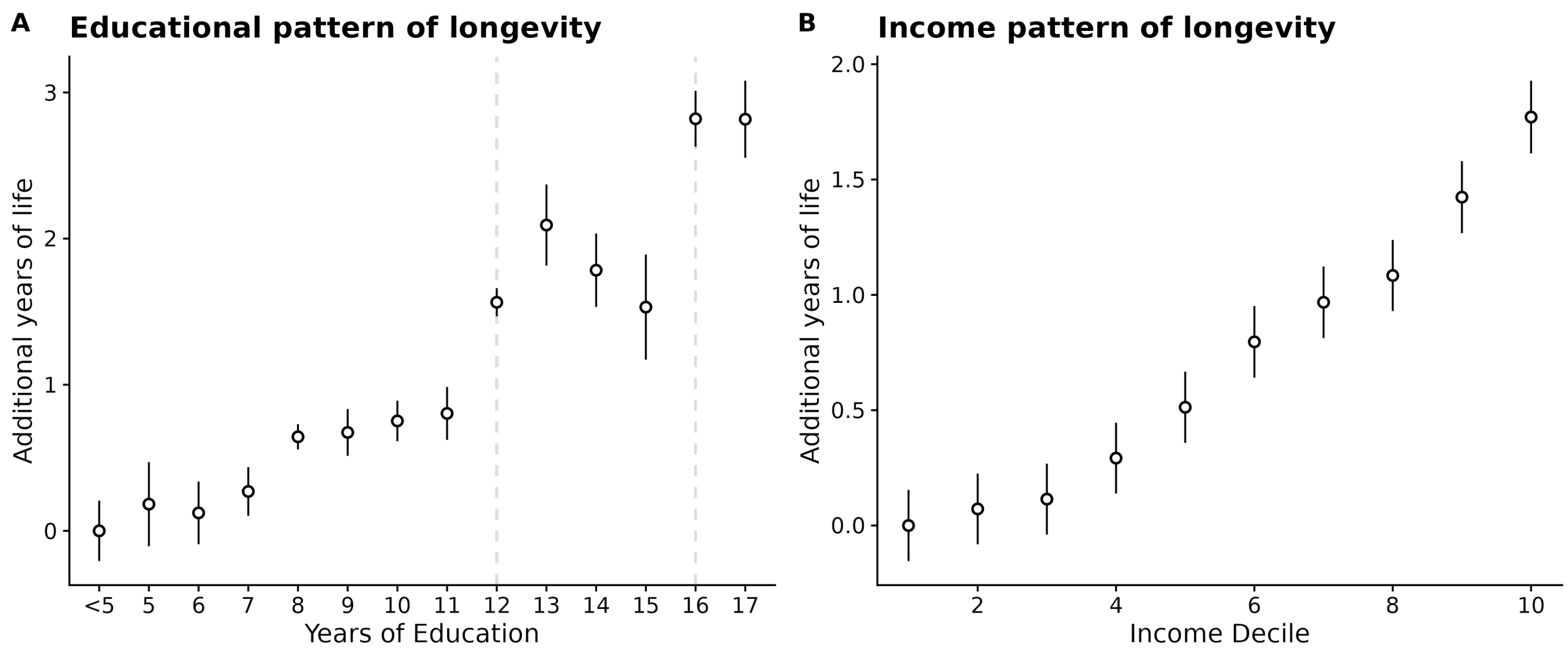

Figure A4: *(A)* Educational and *(B)* income gradients of longevity, calculated for individuals born in 1910. Error bars represent 95% uncertainty intervals.

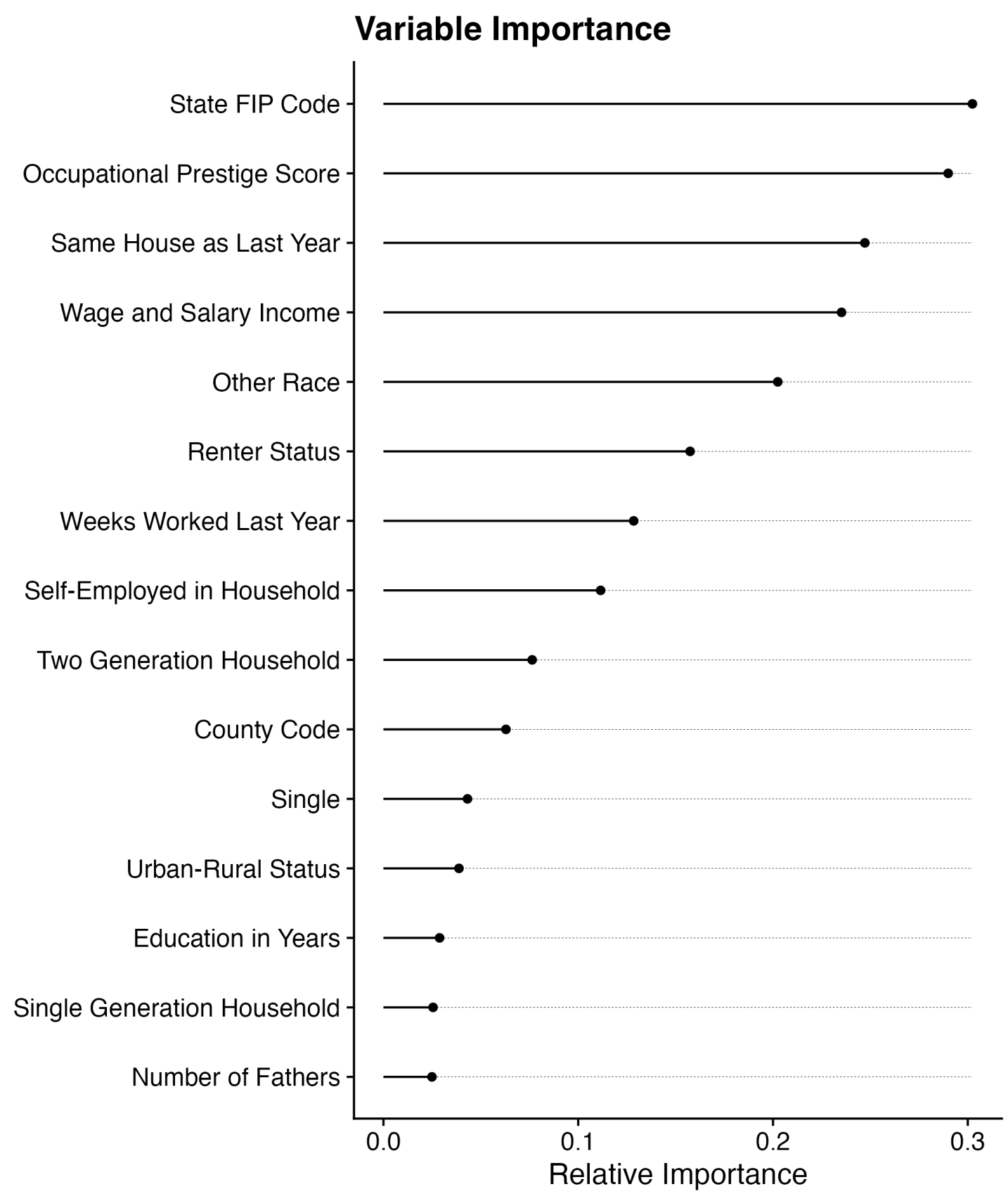

Figure A5: Variable importance for our main Superlearner model using a permutation approach. Specifically, variable importance is measured as the reduction in predictive accuracy (mean squared error) attributable to each variable if that variable was randomly permuted. This offers a different perspective, but it is sensitive to collinearity: highly correlated variables may mask each other's importance.

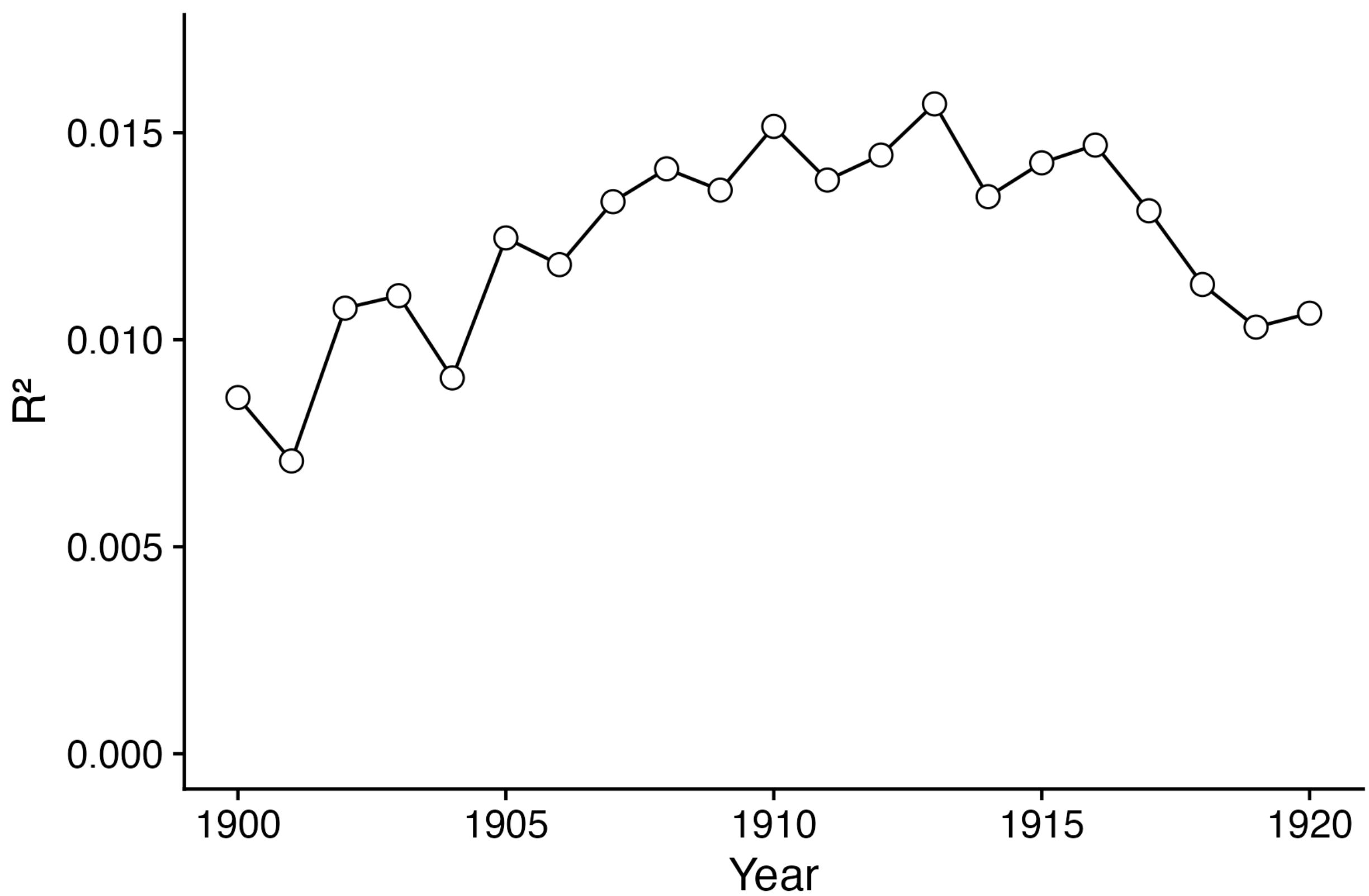

Figure A6: The estimated R^2 values from a replication of the main CenSoc-DMF prediction exercise, repeated separately for each birth cohort from 1900 to 1920. Performance is based on a gradient boosted model (GBM), our top-performing discrete machine-learning algorithm. We caution against overinterpreting trends, as each cohort will have: (1) covariates measured at a different point of the life course; (2) mortality observed for a different range of ages; and (3) cohort-specific exposures across the life course. In particular, directly testing whether covariates observed at earlier or later stages of the life course are more predictive is challenging because differences in predictive performance are also influenced by the different ages for which mortality is observed (e.g., the 1900 cohort has mortality observed from ages 75 to 105, while the cohort of 1920 has mortality observed from ages 55 to 85). Differences in predictive performance among cohorts likely reflect differences in age exposure rather than the intrinsic informativeness of later-life covariates. We include this figure to emphasize that predictability is low across all cohorts considered.

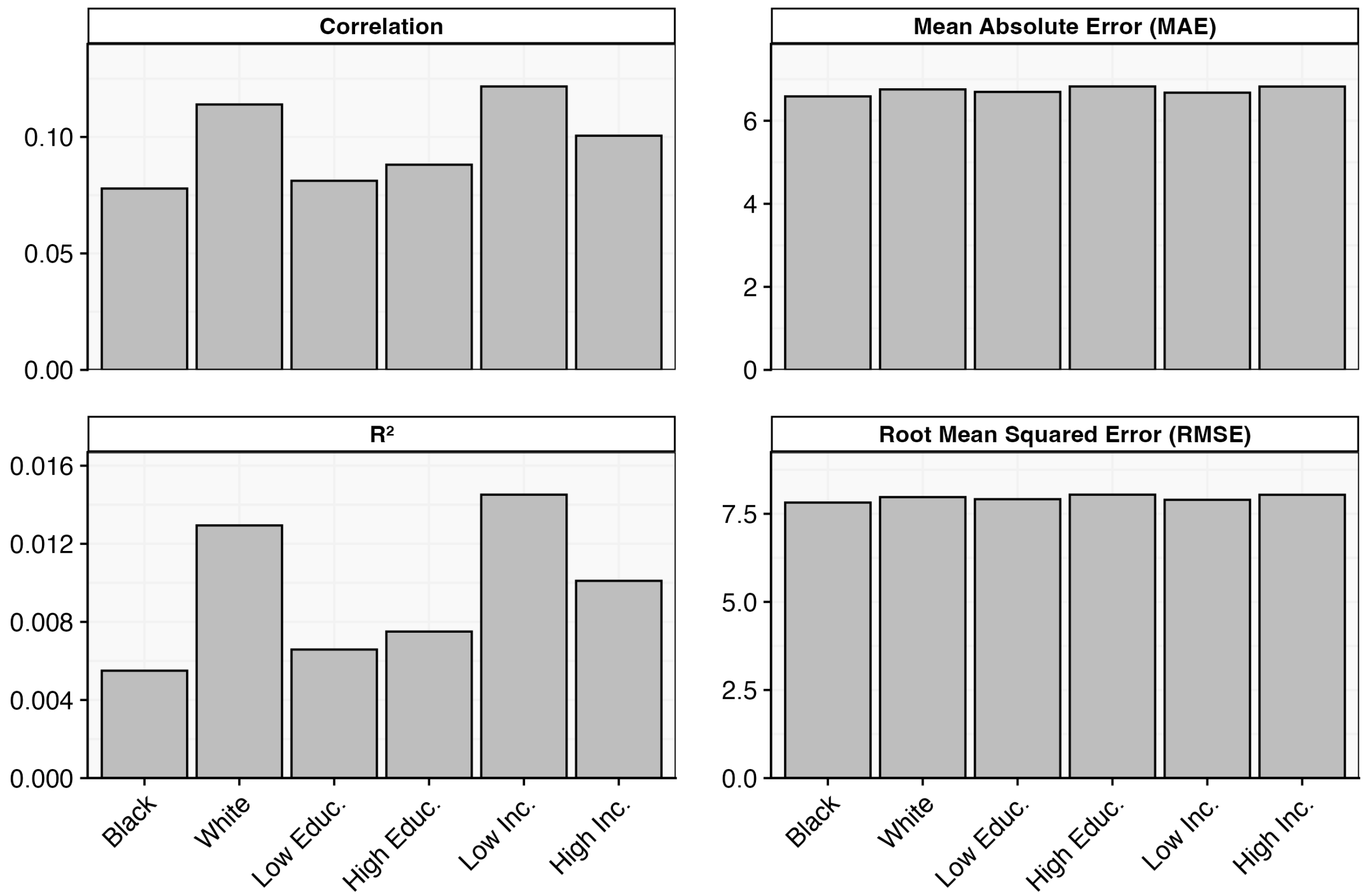

Figure A7: Performance metrics of the Superlearner algorithm by demographic and socioeconomic subgroups. Metrics include correlation, R^2, mean absolute error (MAE), and root mean squared error (RMSE); higher correlation and R^2 indicate better fit, while lower MAE and RMSE indicate lower prediction error. High and low education refer to educational attainment above or below sample median. High and low income refer to wage and salary income above or below sample median.

F REFORM Checklist

In order to promote transparency, we completed the REFORM checklist (Kapoor et al., 2024). The REFORM checklist, developed collaboratively by 19 researchers across the social, computer, and physical sciences, is a resource for promoting transparency and reproducibility in machine learning science.

F.1 Study Goals

1a. *State the population or distribution about which the scientific claim is made.*

In the primary analysis, this study makes claims about the predictability of lifespan using sociodemographic characteristics for the population of U.S. men born in 1910 dying between 1975 and 2005.

1b. *Describe the motivation for choosing this population or distribution.*

We focus on this population for several reasons. First, we are interested in mortality prediction in the United States, a country with well-documented group-level disparities in mortality. Second, we focus on these specific cohorts and age ranges due to data availability limitations—our mortality records only have complete coverage from 1975 to 2005. Importantly, our main analysis is restricted to men, as surname changes for women during marriage prevent accurate record linkage of women. Finally, we are interested in individuals who are adults when enumerated in the 1940 Census, as we want to measure covariates in early adulthood.

1c. *Describe the motivation for the use of ML methods in the study.*

The use of ML methods in this study is appropriate as our primary scientific interest

is assessing the predictability of individual-level mortality. Therefore, our interest is in maximizing the predictive power, rather than the explanatory power, of our models.

F.2 Computational Reproducibility

2a. *Describe the dataset used for training and evaluating the model and provide a link or DOI to uniquely identify the dataset.*

We use the publicly-available V3 CenSoc-DMF dataset (Goldstein et al., 2023).

2b. *Provide details about the code used to train and evaluate the model and produce the results reported in the paper along with link or DOI to uniquely identify the version of the code used.*

The models were fit using the SL3 package in R (Coyle et al., 2021). Code is available from a dedicated OSF repository [link].

2c. *Describe the computing infrastructure used.*

All computations were carried out on a 2023 MacBook Pro with an Apple M2 Pro chip, 16GB memory, and Sonoma 14.1 operating system.

2d. *Provide a README file which contains instructions for generating the results using the provided dataset and code.*

A README file is available as part of our replication package available on our OSF repository.

2e. *Provide a reproduction script to produce all results reported in the paper.*

A replication script is available on a dedicated OSF repository.

F.3 Data Quality

3a. *Describe source(s) of data, separately for the training and evaluation datasets, along with the time when the dataset(s) are collected, the source and process of ground-truth annotations, and other data documentation.*

The data are from the CenSoc project, which links the 1940 Census and Social Security mortality records (Breen, Osborne and Goldstein, 2023). Both our cohort and period datasets are split into a 75% training and 25% holdout dataset.

3b. *State the distribution or set from which the dataset is sampled (i.e., the sampling frame).*

The full sampling frame is all people enumerated in the 1940 Census who were successfully linked to death records between 1975 and 2005. For the cohort analysis, the data are first restricted to the birth cohort of 1910. For the period analysis, we take a random sample of 100,000 individuals between the ages of 54 and 95 in 1975. We take a random sample to minimize computational burden.

3c. *Justify why the dataset is useful for the modeling task at hand.*

This individual-level dataset contains many sociodemographic characteristics and age of death. In addition, the large size of this dataset and longitudinal structure allows us to investigate prediction from both a cohort and a period perspective.

F.4 Data Preprocessing

4a. *Describe whether any samples are excluded with a rationale for why they are excluded.*

For both cohort and period analyses, we exclude individuals with missing variables. We are interested in prediction for individuals with complete information on sociode-

mographic characteristics.

4b. *Describe how impossible or corrupt samples are dealt with.*

There are no impossible or corrupt samples.

4c. *Describe all transformations of the dataset from its raw form to the form used in the model, for instance, treatment of missing data and normalization—preferably through a flow chart.*

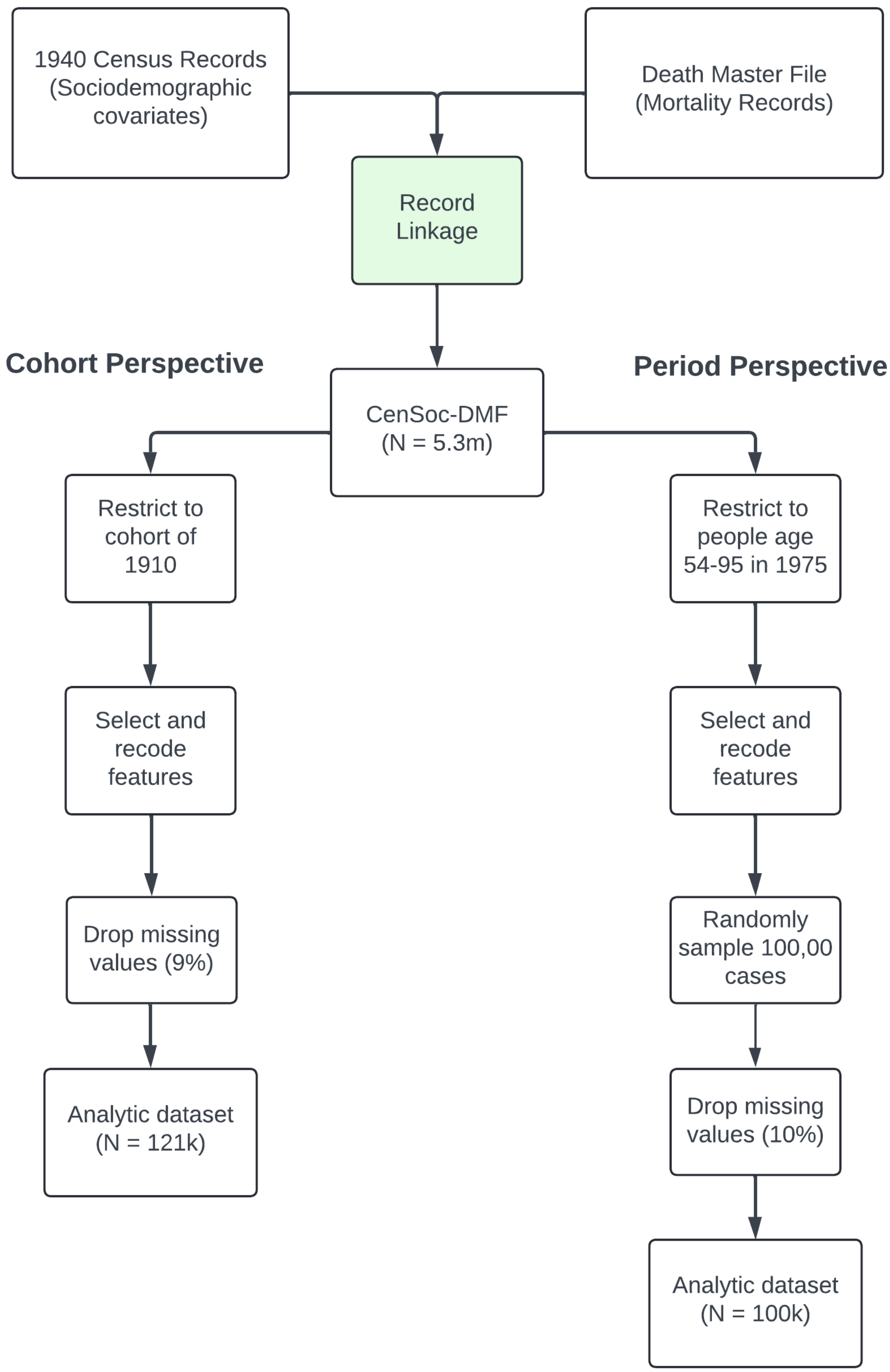

Figure A8: Data processing flowchart.

F.5 Modeling

5a. *Describe, in detail, all models trained.*

Our superlearner includes the following algorithms: gradient boosting machines, linear model, extreme gradient boosting, random forest regression, support vector machine, lasso regression, and multivariate adaptive regression splines (MARS). The model is fit using the SL3 package, an R package for fitting a Superlearner, a type of ensemble or "stacked" algorithm. The metalearner, which combines prediction from the set of discrete algorithms, is a non-negative ordinary least squares model.

5b. *Justify the choice of model types implemented.*

The key advantage of an ensemble learner with many different algorithms is that it reduces the need to rely on a given algorithm. Our candidate algorithms for our Superlearner were picked based on popularity and strong performance in similar prediction tasks.

5c. *Describe the method for evaluating the model(s) reported in the paper, including details of train-test splits or cross-validation folds.*

To assess the model performance, we randomly partition the data into a 75% training and 25% holdout training sets. Within the training dataset, the Superlearner algorithm uses 10-fold cross-validation to pick the best combination of algorithms.

5d. *Describe the method for selecting the model(s) reported in the paper.*

To select the models used in the Superlearner, we picked common machine learning algorithms that had performed well in other settings. We picked a diverse set of

algorithms to include in our ensemble per best practice (Phillips et al., 2023)

5e. *For the model(s) reported in the paper, specify details about the hyperparameter tuning.*

We conduct hyperparameter tuning to optimize the random forest and support vector machine algorithms. For random forest, we use a defined grid of hyperparameters, which includes the number of trees (100, 500, 1000), the number of variables to possibly split at each node (2, 3, 4), the minimum size of tree nodes (1, 5, 10), and the fraction of the data sampled for growing the trees (0.65, 0.8, 1). For Support Vector Machines (SVM), we use a structured search over a grid of two key hyperparameters: the cost parameter (0.1, 1, 10) and the gamma parameter (0.01, 0.1, 1). These parameters control the penalty of the error term and the influence of a single training example, respectively.

5f. *Justify that model comparisons are against appropriate baselines.*

Not applicable.

F.6 Data Leakage

6a. *Justify that pre-processing and modeling steps only use information from the training dataset (and not the test dataset).*

The pre-processing and modeling steps only use information available in the training dataset. The holdout data are exclusively used for prediction.

6b. *Describe methods used to address dependencies or duplicates between the training and test datasets.*

There are no dependencies or duplicates between the training and test (holdout) datasets.

6c. *Justify that each feature or input used in the model is legitimate for the task at hand and does not lead to leakage.*

No features or input in this model will lead to data leakage based on our study design. We randomly split our sample into a test and a holdout sample, and never use holdout data to train our models.

F.7 Metrics and Uncertainty

7a. *State all metrics used to assess and compare model performance. Justify that the metric used to select the final model is suitable for the task.*

For our cohort analysis predicting individual age of death, we used R^2 and mean absolute error (MAE). These metrics were selected as they are among the most popular metrics used in mortality prediction settings, allowing for the direct comparison to other studies. Further, these metrics are intuitive and straightforward to interpret.

7b. *State uncertainty estimates and give details of how these are calculated.*

We did not calculate estimates of uncertainty.

7c. *Justify the choice of statistical tests (if used) and a check for the assumptions of the statistical test.*

We did not perform any statistical tests.

F.8 Generalizability and Limitations

8a. *Describe evidence of external validity.*

Our findings align with past research on relative contribution of within-group differences and between-group differences to longevity (Caswell, 2009, 2023; Vaupel, 1988). However, assessing the predictability of mortality in other countries, ages, and time periods is an important direction for future research.

8b. *Describe contexts in which the authors do not expect the study's findings to hold.*

This study focuses on core sociodemographic characteristics within the context of a wealthy, developed country. We expect the broad patterns observed here to generalize across similar high-income settings with established data infrastructure systems and high social stratification. However, the extent to which these results would hold in low- and middle-income countries, where mortality patterns, data quality, and the salience of social determinants may differ, remains an open question.
